# Effects of Common Polymorphisms rs11614913 in *miR-196a2* and rs2910164 in *miR-146a* on Cancer Susceptibility: A Meta-Analysis

**DOI:** 10.1371/journal.pone.0020471

**Published:** 2011-05-26

**Authors:** Wei Xu, Jijun Xu, Shifeng Liu, Bo Chen, Xueli Wang, Yan Li, Yun Qian, Weihong Zhao, Jianqing Wu

**Affiliations:** 1 Department of Geriatrics, The First Affiliated Hospital of Nanjing Medical University, Nanjing, China; 2 College of Economics and Management, Nanjing Agricultural University, Nanjing, China; Ohio State University Medical Center, United States of America

## Abstract

**Background:**

MicroRNAs regulate gene expression at the post-transcriptional level and involved in diverse biological and pathological processes, including tumorigenesis. Rs11614913 in *miR-196a2* and rs2910164 in *miR-146a* are shown to associate with increased/decreased cancer risk. We performed a meta-analysis to systematically summarize the possible association.

**Methodology/Principal Findings:**

We assessed published studies of the association between these microRNA polymorphisms and cancer risk from eleven studies with 16,771 subjects for *miR-196a2* and from ten studies with 15,126 subjects for *miR-146a*. As for rs11614913, the contrast of homozygote (TT *vs* CC: OR = 0.92, 95% CI = 0.85–0.99, *P*_heterogeneity_ = 0.45), allele (T *vs* C: OR = 0.96, 95% CI = 0.92–0.99, *P*_heterogeneity_ = 0.61) and recessive model (OR = 0.90, 95% CI = 0.84–0.97, *P*_heterogeneity_ = 0.50) produced statistically association. Subgroup analysis by ethnicity, statistically significantly decreased cancer risks were found among Asians for allele contrast (OR = 0.95, 95% CI = 0.90–0.99, *P*_heterogeneity_ = 0.74) and the recessive genetic model (OR = 0.90, 95% CI = 0.82–0.98, *P*_heterogeneity_ = 0.85). According to subgroup analysis by tumor types, the protective effect of C/T polymorphism was only found in breast cancer under allele contrast (T *vs* C: OR = 0.94, 95% CI = 0.88–0.99, *P*_heterogeneity_ = 0.26). For rs2910164, no significant associations were found among overall analysis model with relatively large heterogeneity. Through the stratified analysis, heterogeneity decreased significantly. In the subgroup analyses by cancer types, the C allele of rs2910164 was associated with protection from digestive cancer in allele contrast (C *vs* G: OR = 0.86, 95% CI = 0.77–0.96, *P*_heterogeneity_ = 0.51).

**Conclusions/Significance:**

Our meta-analysis suggests that the rs11614913 most likely contributes to decreased susceptibility to cancer, especially in Asians and breast cancer. Besides, the C allele of the rs2910164 might be associated with a protection from digestive cancer.

## Introduction

MicroRNAs (miRNAs) are a class of naturally occurring, small non-coding, single-stranded RNA molecules that form base-pairs with target mRNAs and negatively regulate their translational efficiency and stability [1]. Bioinformatics study indicates that a single miRNA can bind to as many as 200 gene targets, and one-third of human genes, including cancer-associated genes, are regulated by miRNAs [2]. A strong link between miRNA and human cancers has been established, as miRNAs have been demonstrated to act as either oncogenes or tumor suppressors. The main molecular mechanism underlies changes in the function of miRNAs in cancer cells seems to be aberrant gene expression [3]. miRNA expression profiles have been reported to be correlated with the etiology, classification, progression, and prognosis of multiple human cancers. Since small variation in expression of a specific miRNA may effect on thousands of target mRNAs and result in diverse functional consequences [4], miRNAs represent ideal candidates for cancer predisposition genes.

Single nucleotide polymorphisms (SNPs) or mutations occurring in the *miRNA* gene region may affect the property of miRNAs through altering miRNA expression and/or maturation [5]. However, the role of genetic variants in miRNAs on cancer susceptibility remains largely unknown. Recently, several reports identified genetic variants in the precursor or mature miRNA sequence of *miR-196a2* (rs11614913 *[Homo sapiens]*, cytosine to thymine, C→T) and *miR-146a* (rs2910164 *[Homo sapiens]*, guanine to cytosine, C→G, http://www.ncbi.nlm.nih.gov/projects/SNP) as possible biomarkers, which were associated with multiple kind of malignant tumors, such as those that occur in the central nervous system [6], head and neck [7], lung [8], [9], esophagus [10], stomach [11], biliary tract [12], liver [13]–[15], breast [16]–[19], ovarian [19], prostate [20] and thyroid [21]. However, the observed associations of these studies were inconsistent and a single study may be too underpowered to detect a possible small effect of the gene polymorphism on cancer, especially when the sample size is relatively small. Hence, we performed a meta-analysis of all eligible studies to derive more precise estimation of the association of *mir-196a2* C/T and *mir-146a* G/C SNP with cancer risks.

## Materials and Methods

### Publication search

We carried out a search in Medline, Embase and Chinese National Knowledge Infrastructure (CNKI) databases, covering all papers published between 1991 and 2011, with a combination of the following keywords: “microRNA/miR-196a2/miR-146a”, “gene”, “polymorphism” and “cancer” (last search was updated on 15 Jan 2011). We evaluated potentially relevant publications by examining their titles and abstracts and all studies matching the eligible criteria were retrieved.

### Inclusion criteria

Studies included in the current meta-analysis had to meet all the following criteria: (a) evaluation of the rs11614913 and/or rs2910164 and cancer risks, (b) use a case-control design, (c) sufficient published data for estimating an odds ratio (OR) with 95% confidence interval (CI).

### Data extraction

Data were independently abstracted in duplicate by two investigators (Xu and Chen) using a standard protocol and data-collection form according to the inclusion criteria listed above. Characteristics abstracted from the studies included the name of first author, publication date, country origin, ethnicity, cancer type, control characteristics, genotyping methods, total number of cases and controls, and numbers of cases and controls with *miR-196a2* C/T and/or *miR-146a* G/C genotypes, respectively. Different ethnicity descents were categorised as Caucasian and Asian.

### Statistical methods

OR corresponding to 95% CI was used to assess the strength of association between *microRNA* polymorphism and cancer. The significance of the pooled OR was determined by the *Z*-test, and *P*<0.05 was considered as statistically significant. For *miR-196a2* C/T, the meta-analysis examined the association between T allele and cancer risk compared with that for C allele (T *vs* C); homozygote TT was contrasted with CC (TT *vs* CC) and recessive (TT *vs* CC+CT) and dominant (TT+CT *vs* CC) models for allele T were also used, so was *miR-146a*. Subgroup analyses were done by racial descent and tumor type.

Heterogeneity in meta-analysis refers to the variation in study outcomes between different studies. Heterogeneity assumption was checked by the chi-square-based *Q*-test [22]. A *P*-value>0.10 for the *Q*-test indicates a lack of heterogeneity among the studies, then the pooled OR estimate of each study was calculated by the fixed-effects model (the Mantel-Haenszel method) [23]. Otherwise, the random-effects model (the DerSimonian and Laird method) [24] was used. Hardy-Weinberg equilibrium (HWE) in the control group was assessed via Fisher's exact test and a *P*-value<0.05 was considered significant. One-way sensitivity analyses were performed to determine whether the assumptions or decisions we have made do in fact have a major effect on the results of the review, namely, a single study in the meta-analysis was deleted each time to reflect the influence of the individual data-set to the pooled OR. Publication bias was assessed by visual inspection of funnel plots in which the standard error of log (OR) of each study was plotted against its log (OR). An asymmetric plot suggests a possible publication bias. Funnel plot asymmetry was assessed by the method of Egger's linear regression test, a linear regression approach to measure funnel plot asymmetry on the natural logarithm scale of the OR. The significance of the intercept was determined by the *t*-test (*P*<0.05 was considered representative of statistically significant publication bias) [25]. All of the statistical analyses were performed with STATA 9.2 (StataCorp, College Station, TX), using two-sided *P*-values.

## Results

### Study characteristics

**Figure 1:**
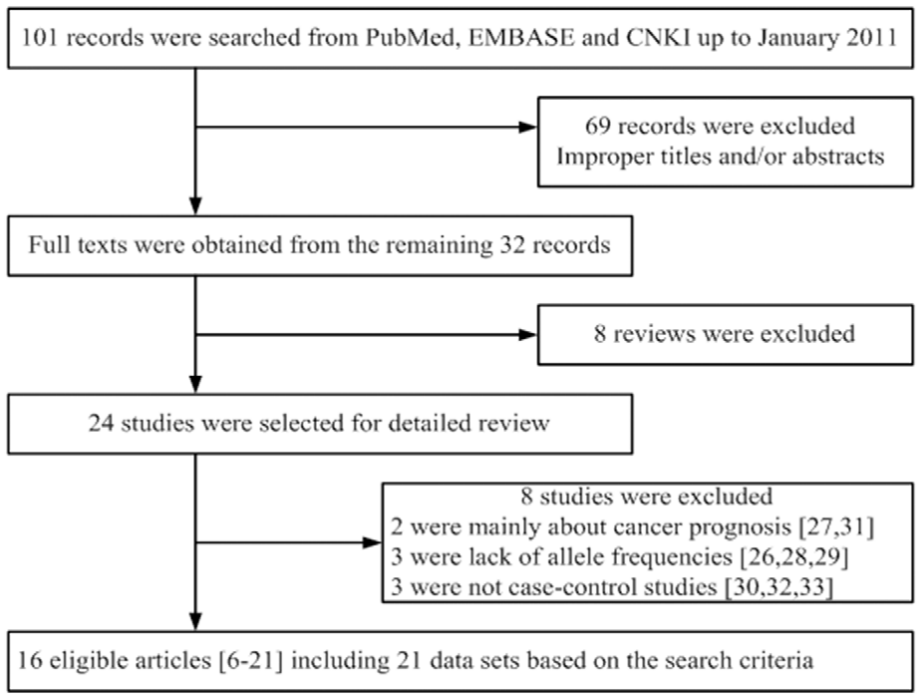
Flow diagram of study identification.

A total of 101 articles were achieved by literature search, from the PubMed, EMBASE and CNKI database, using different combination of key terms. As shown in Figure 1, twenty-four eligible studies were retrieved for detailed evaluation. During the extraction of data, eight articles [26]–[33] were excluded, because they did not provide rs11614913 or rs2910164 allele frequencies needed for OR calculation, lack of control or their contents mainly associated with cancer prognosis and therapy, leaving 16 eligible articles [6]–[21] including 21 data sets based on the search criteria. Five studies [7], [8], [12], [16], [17] sorted the data about two kinds of *microRNAs*, therefore, each group in these studies was considered separately for pooling analyses. The characteristics of selected studies are summarized in Table 1. A total of 11 studies [6]–[9], [11]–[14], [16], [17], [19] involving 7,992 cases and 8,849 controls were ultimately analyzed for rs11614913, ten studies [7], [8], [10], [12], [15]–[17], [19]–[21] involving 7,183 cases and 7,943 controls for rs2910164. As for rs11614913, there were four studies of Caucasians [7], [12], [17], [19] and seven studies of Asians [6], [8], [9], [11], [13], [14], [16]. For rs2910164, there were 5 groups of Asians [8], [10], [15], [16], [20] and 5 of Caucasians [7], [12], [17], [19], [21]. The controls of all studies mainly came from healthy population and matched for sex and age. The distribution of genotypes in the controls did not deviate from HWE.

**Table 1:** Main characteristics of all studies included in the meta-analysis.

Author, year	Country	Ethnicity	Cancer type	Gene type	Genotyping method	No. (cases/controls)	Case (%)			Control (%)		
							CC/GG	CT/GC	TT/CC	CC/GG	CT/GC	TT/CC
Hu 2008	China	Asian	Breast	rs11614913	PCR-RFLP	1009/1093	239(24.0)	483(48.0)	287(28.0)	218(20.0)	517(47.0)	358(33.0)
				rs2910164	PCR-RFLP	1009/1093	165(17.0)	515(51.0)	329(33.0)	180(17.0)	551(50.0)	362(33.0)
Xu 2008	China	Asian	Liver	rs2910164	PCR-RFLP	479/504	80(16.7)	241(50.3)	158(33.0)	58(11.5)	249(48.4)	197(39.1)
Krystian 2008	USA	Caucasian	PTC	rs2910164	Taqman	608/901	305(50.2)	287(47.2)	16(2.6)	526(58.4)	320(35.5)	55(6.1)
Tian 2009	China	Asian	Lung	rs11614913	PCR-RFLP	1058/1035	253(23.9)	512(48.4)	293(27.7)	209(20.2)	519(50.1)	307(29.7)
				rs2910164	PCR-RFLP	1058/1035	360(34.0)	510(48.2)	188(17.8)	364(35.2)	502(48.5)	169(16.3)
Hoffman 2009	USA	Caucasian	Breast	rs11614913	massARRAY	441/479	181(42.5)	209(49.1)	36(8.4)	166(35.6)	229(49.1)	71(15.3)
Srivastava 2010	India	Caucasian	Gallbladder	rs11614913	PCR-RFLP	230/230	136(59.1)	75(32.6)	19(8.3)	119(51.7)	95(41.3)	16(7.0)
				rs2910164	Taqman	230/230	138(61.6)	81(36.2)	5(2.2)	129(56.1)	90(39.1)	11(4.8)
Min 2010	Korean	Asian	Lung	rs11614913	PCR+MCS	654/640	187(28.6)	305(46.6)	162(24.8)	155(24.2)	300(46.9)	185(28.9)
Li 2010	China	Asian	Liver	rs11614913	PCR-RFLP	310/222	78(25.2)	150(48.4)	82(26.4)	42(18.9)	102(46.0)	78(35.1)
Dou 2010	China	Asian	Glioma	rs11614913	PCR-LDR	643/656	111(17.3)	343(53.3)	189(29.4)	143(21.8)	305(46.5)	208(31.7)
Qi 2010	China	Asian	Liver	rs11614913	PCR-LDR	361/391	82(22.7)	179(49.6)	100(27.7)	92(23.5)	197(50.4)	102(26.1)
Catucci 2010	Italy	Caucasian	Breast	rs11614913	PCR-RFLP	1471/1243	334(45.5)	330(43.9)	87(11.6)	532(42.8)	550(44.3)	161(13.0)
				rs2910164	PCR-RFLP	754/1243	409(54.2)	286(37.9)	59(7.8)	650(52.3)	520(41.8)	73(5.9)
	Germany	Caucasian	Breast	rs11614913	PCR-RFLP	1101/1196	432(39.2)	512(46.5)	157(14.3)	584(39.0)	396(46.5)	216(14.4)
				rs2910164	PCR-RFLP	813/904	459(56.0)	304(37.8)	50(6.2)	536(59.3)	318(35.2)	50(5.5)
Liu 2010	USA	Caucasian	SCCHN	rs11614913	PCR-RFLP	1109/1130	350(31.6)	565(50.9)	194(17.5)	383(33.9)	545(48.2)	202(17.9)
				rs2910164	PCR-RFLP	1109/1130	630(56.8)	411(37.1)	68(6.1)	655(58.0)	405(35.8)	70(6.2)
Peng 2010	China	Asian	Gastric	rs11614913	PCR-RFLP	213/213	76(35.7)	94(44.1)	43(20.2)	56(26.3)	107(50.2)	50(23.5)
Xu 2010	China	Asian	Prostate	rs2910164	PCR-RFLP	251/280	68(27.1)	135(53.8)	48(19.1)	54(19.3)	150(53.6)	76(27.1)
Guo 2010	China	Asian	ESCC	rs2910164	SNaPshot	444/468	234(52.7)	190(42.8)	20(4.5)	206(44.0)	220(47.0)	42(9.0)
Pastrello 2010	Italy	Caucasian	Mix	rs2910164	Sequencing	101/155	60(59.0)	36(36.0)	5(5.0)	90(58.0)	59(38.0)	6(4.0)

PTC: papillary thyroid carcinoma. ESCC: esophageal squamous cell carcinoma. SCCHN: cell carcinoma of head and neck.

### Meta-analysis results

The allele frequencies were calculated for controls from the corresponding genotype distributions. The two variant alleles had the higher representations among controls of Asian descent (0.544 for T allele in rs11614913, 0.498 for C allele in rs2910164) than in controls of Caucasian descent (0.375 for T allele in rs11614913, 0.246 for C allele in rs2910164) respectively.

**Figure 2:**
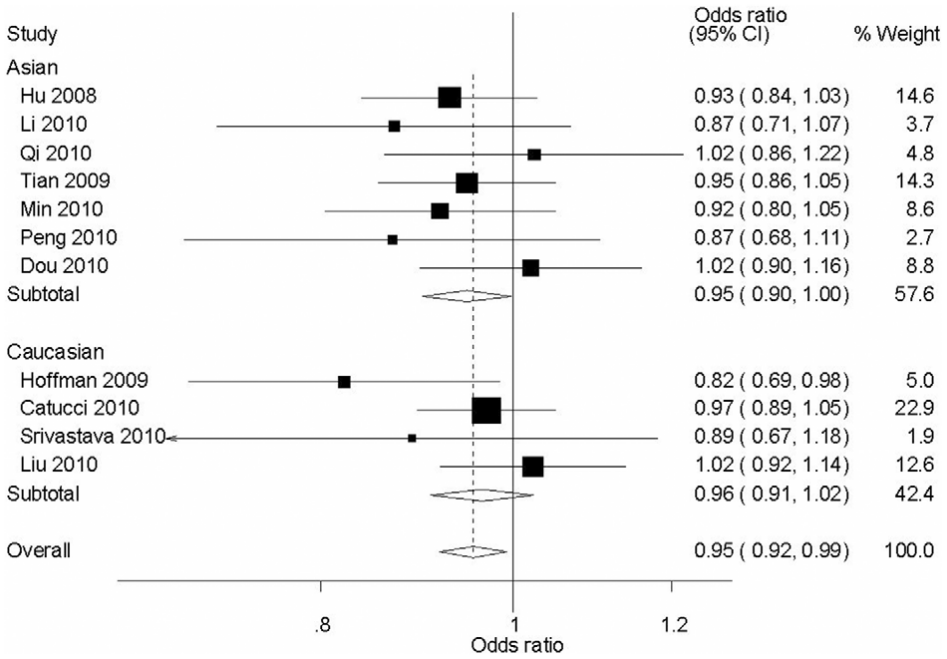
Forest plot of cancer risk associated with rs11614913 under allele contrast (T *vs* C) in different ethnicity. The squares and horizontal lines correspond to the study-specific OR and 95% CI. The area of the squares reflects the study specific weight (inverse of the variance). The diamond represents the pooled OR and 95% CI.

**Figure 3:**
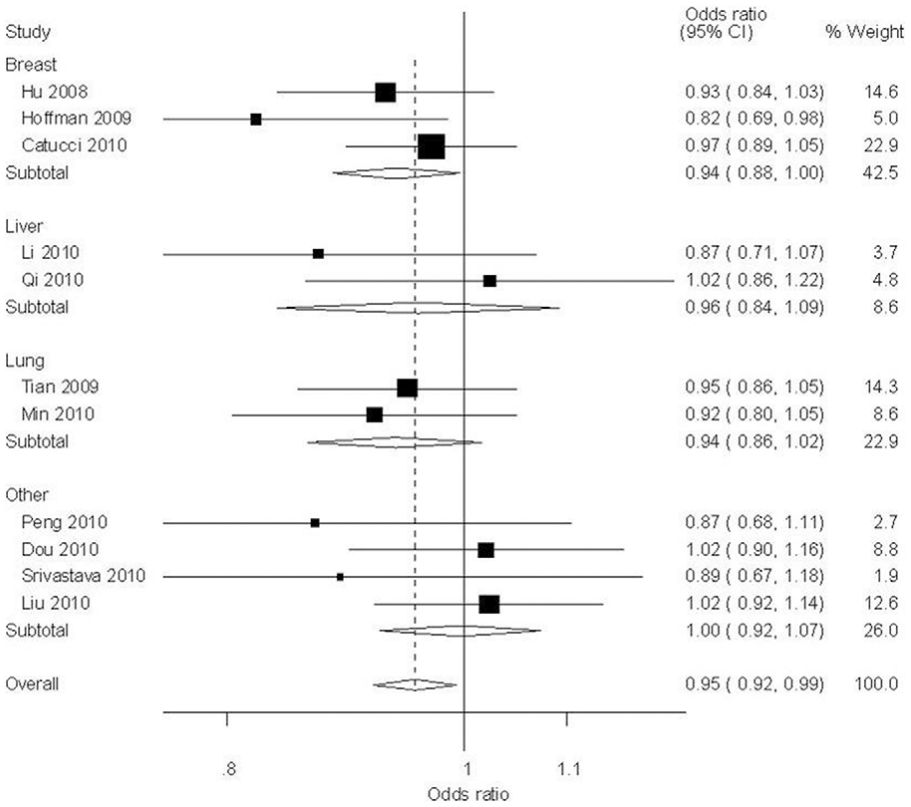
Forest plot of cancer risk associated with rs11614913 under allele contrast (T *vs* C) in different types of cancers. The squares and horizontal lines correspond to the study-specific OR and 95% CI. The area of the squares reflects the study specific weight (inverse of the variance). The diamond represents the pooled OR and 95% CI.

The overall OR with its 95% CI showed statistically association between the rs11614913 and the reduced risks of cancers (TT *vs* CC: OR = 0.92, 95% CI = 0.85–0.99, *P*_heterogeneity_ = 0.45; T *vs* C: OR = 0.96, 95% CI = 0.92–0.99, *P*_heterogeneity_ = 0.61 and the recessive model: OR = 0.90, 95% CI = 0.84–0.97, *P*_heterogeneity_ = 0.50). In the subgroup analysis by ethnicity, statistically significantly decreased cancer risks were found among Asians for allele contrast (OR = 0.95, 95% CI = 0.90–0.99, *P*_heterogeneity_ = 0.74, Fig. 2) and the recessive model (OR = 0.90, 95% CI = 0.82–0.98, *P*_heterogeneity_ = 0.85). In the tumor type subgroup analysis, T allele had an effect of decreasing the risk of breast (T *vs* C: OR = 0.94, 95% CI = 0.88–0.99, *P*_heterogeneity_ = 0.26, Fig. 3).

For rs2910164, five kinds of genetic models did not produce significant association among all studies with relatively large heterogeneity (*P*_heterogeneity_ = 0.03–0.41). Through stratified analyses, the heterogeneity of the subgroup significantly reduced. In the stratified analysis by racial descent, no significant risks were found among Asians and Caucasians. Carriage of the C variant of rs2910164 was found to be associated with protection from digestive cancer (C *vs* G: OR = 0.86, 95% CI = 0.77–0.96, *P*_heterogeneity_ = 0.51) in the subgroup analyses by cancer types. No significant associations were found in breast cancer and other subgroups. The data are shown in Table 2.

**Table 2:** Main results of pooled ORs and stratification analysis of the *miR-196a2* C/T (rs11614913) and *miR-146a* G/C (rs2910164) functional polymorphisms on cancer risk in the meta-analysis.

*miR-196a2*		TT *vs* CC	CT *vs* CC	T *vs* C	Dominant model	Recessive model
C/T	N	OR (95% CI) P*h*	OR (95% CI) P*h*	OR (95% CI) P*h*	OR (95% CI) P*h*	OR (95% CI) P*h*
Total	11	0.92(0.85–0.99)0.45	0.98(0.93–1.03)0.87	0.96(0.92–0.99)0.61	0.97(0.93–1.02)0.93	0.90(0.84–0.97)0.50
Cancer types						
Breast	3	0.84(0.78–1.05)0.08	0.97(0.89–1.06)0.82	0.94(0.88–0.99)0.26	0.96(0.89–1.03)0.73	0.83(0.77–1.03)0.07
Lung	2	0.88(0.75–1.04)0.74	0.94(0.83–1.07)0.99	0.94(0.86–1.02)0.71	0.95(0.86–1.05)0.91	0.91(0.78–1.05)0.57
Liver	2	0.93(0.72–1.19)0.29	0.98(0.80–1.19)0.71	0.96(0.84–1.09)0.24	0.97(0.82–1.15)0.60	0.92(0.72–1.16)0.15
Others	4	1.01(0.87–1.18)0.69	1.02(0.92–1.04)0.29	0.99(0.92–1.07)0.53	1.00(0.93–1.02)0.48	0.95(0.83–1.10)0.87
Ethnicity						
Caucasian	4	0.94(0.82–1.07)0.10	0.99(0.91–1.07)0.51	0.96(0.91–1.02)0.21	0.98(0.91–1.05)0.52	0.92(0.81–1.04)0.09
Asian	7	0.91(0.82–1.00)0.75	0.97(0.89–1.05)0.84	0.95(0.90–0.99)0.74	0.97(0.91–1.03)0.91	0.90(0.82–0.98)0.85

*miR-146a*		CC *vs* GG	CG *vs* GG	C *vs* G	Dominant model	Recessive model
G/C	N	OR (95% CI) P*h*	OR (95% CI) P*h*	OR (95% CI) P*h*	OR (95% CI) P*h*	OR (95% CI) P*h*
Total	10	0.89(0.75–1.05)0.03	1.01(0.95–1.07)0.41	0.94(0.88–1.01)0.04	0.96(0.89–1.04)0.06	0.86(0.73–1.01)0.03
Cancer types						
Breast	2	1.07(0.90–1.26)0.28	0.99(0.90–1.09)0.83	0.92(0.78–1.08)0.23	0.91(0.75–1.10)0.03	0.99(0.86–1.14)0.90
Digestive	3	0.63(0.39–1.03)0.10	0.89(0.77–1.04)0.93	0.86(0.77–0.96)0.51	0.89(0.78–1.02)0.73	0.66(0.44–1.01)0.15
Others	5	0.88(0.68–1.15)0.13	1.06(0.97–1.16)0.24	1.02(0.95–1.09)0.45	1.04(0.96–1.13)0.46	0.83(0.61–1.15)0.02
Ethnicity						
Caucasian	5	0.89(0.62–1.26)0.07	1.04(0.96–1.13)0.19	0.95(0.84–1.08)0.04	0.97(0.82–1.15)0.01	0.80(0.56–1.15)0.05
Asian	5	0.87(0.70–1.07)0.05	0.97(0.89–1.06)0.80	0.94(0.86–1.02)0.12	0.97(0.72–1.04)0.63	0.87(0.72–1.06)0.05

N indicates number of studies involved; Ph: P-value of Q-test for heterogeneity test.

### Bias diagnostics

Begg's funnel plot and Egger's test were performed to assess the publication bias of the literature. The shapes of the funnel plot for the comparison of the T allele and the C allele of rs11614913 seemed approximately symmetrical, and Egger's test did not show any evidence of publication bias (*t* = 0.54, df = 10, *P* = 0.60, Figure S1). So did *miR-146a* (*t* = 0.56, df = 9, *P* = 0.59, Figure S2).

### Sensitivity analysis

A single study involved in the meta-analysis was deleted each time to reflect the influence of the individual data set to the pooled ORs. The corresponding pooled ORs were not materially altered for rs11614913. For rs2910164, one study (Catucci et al. [18]) was considered as the main cause of heterogeneity. After exclusion of this study, the heterogeneity no longer existed, but still reached a negative association (Table S1).

## Discussion

Much research effort has been directed toward understanding the role of SNPs present in precursor and mature miRNA and their influences on susceptibility and progression of various diseases. Although hundreds of SNPs located in the regions of miRNA genes have been identified, most of them do not affect miRNA expression and function. However, two common SNPs, rs11614913 in *miR-196a2* and rs2910164 in *miR-146a*, were found to alter miRNA expression and lead to alter regulation of target mRNAs [6]–[21], [26]–[33]. In the current meta-analysis, we summarized the data on the association between the *miR-196a2* or *miR-146a* functional polymorphism and cancer risks. We found a significantly protective effect of rs11614913 T variant for cancer (Table 2), especially, in the subgroups of Asians (Figure 2) and breast cancers (Figure 3). Besides, the C allele of the rs2910164 might be associated with protection from digestive cancer (Table 2).

In the subgroup analysis, our data indicated rs11614913 leaded to reduced incidence of breast cancer. Comparing expression levels of the mature miR-196a among *miR-196a2* genotypes, Hu et al. [27] observed significantly lower expression of miR-196a in non-small cell lung tumor samples with CC genotype compared to CT and TT individuals, without differential expression of pri-miRNAs and pre-miRNAs based on genotype. In a separate report, Hoffman et al. [17] showed increased expression of mature miR-196a in breast cancer cells transfected with pre-miR-196a-C vector compared with cells transfected with pre-miR-196a-T, but did not observe differential expression of the pre-miRNA, suggesting that *miR-196a2* genotype may result in altered processing of the pre-miR. While Christensen et al. [26] did not detect significantly different miRNA expression by *miR-196a2* genotype in either normal head and neck tissues or tumors. These conflicting results showed a wide range of *miR-196a* expression across different normal tissues, tumor types, and between tumor and normal tissues from common sites.

Some studies have shown that the cleavage of mRNAs of *HOX* gene clusters was partly miR-196a-directed [34]. Abnormal *HOX* gene expression has been associated with breast cancer and *HOXD10* was recently identified as a gene target for the initiation of breast cancer invasion and metastasis [35]. Furthermore, LSP1 and TOX3 mRNAs were targeted by miR196a-3p and miR196a-5p respectively, and these genes were also identified as novel breast cancer susceptibility markers. By other evidence *in vitro*, Hoffman et al. [17] corroborated down-regulation of tumor suppressors (*HOXB2*, *HOXB3*, *HOXB13*, *HOXB5*, *GADD45G*, *INHBB*) and up-regulation of oncogenes (*TP63*, *S100A8*, *S100A9*) by introduction of pre-miR196a-C would be consistent with overall oncogenic activity, and the diminished regulatory activity of pre-miR196a-T is consistent with a protective role for the T allele of the SNP in breast cancer cells.

Rs2910164 in the *miR-146a* gene is located in the stem region opposite to the mature miR-146 sequence and results in a change from G:U pair to C:U mismatch in the stem structure of miR-146a precursor. In two separate studies, Xu et al. [15] and Jazdzewski et al. [21] reported that the G-allelic miR-146a precursor displayed increased production of mature miR-146a compared with the C-allelic one in cell model system and then the miR-146a could obviously promote cell proliferation and colony formation. Jazdzewski et al. [21] also found that rs2910164 in *miR-146a* could affect target mRNA binding. In this meta-analysis, significant association was found about this polymorphism with cancer risk only in the digestive cancer subgroup, and further evaluations were warranted to confirm these results.

One important property of the gene polymorphisms is that their incidence can vary substantially between different racial or ethnic populations. In this meta-analysis, we found highly significant differences in the prevalence of the rs11614913 T allele and rs2910164 C allele among controls of Asian (0.544, 0.498, respectively) and Caucasian (0.375, 0.246, respectively). In the subgroup analysis by ethnicity, the association between T variant genotypes of rs11614913 and reduced risks of cancer was significant only in Asians but not in Caucasians, suggesting genetic diversity among different ethnicities.

Caution must be made about the interpretation of the results because of the relatively large heterogeneity in rs2910164 studies. In the subgroup analyses stratified by tumor site and racial descent respectively, it can be found that the heterogeneity of the subgroup reduced significantly. Therefore, it can be presumed that the relatively large heterogeneity mainly results from differences of ethnicity and tumor types. Simultaneously, the heterogeneity may also have been caused by the differences in the selection of subjects, such as familial breast cancer cases negative for disease-causing mutation in Catucci's research [18]. Publication bias may exist, because only published studies were included in this meta-analysis, although the result for publication bias was not statistically significant. Finally, lack of the original data of the reviewed studies limited our further evaluation of potential interactions, because the interactions between gene-to-gene and gene-to-environment may modulate cancer risk.

In summary, this meta-analysis supports that the rs11614913 in *miR-196a2* most likely contributes to decreased susceptibility to cancer, especially in the subgroup of Asians and breast cancer. Besides, the C allele of rs2910164 in *miR-146a* might be associated with protection from digestive cancer. Larger well-designed studies with subjects of the same ethnic background and tissue-specific biochemical and biological characterization are warranted to validate these findings.

## Supporting Information

Figure S1 **Funnel plot of publication bias in rs11614913 studies.** Log OR is plotted versus standard error for each of studies in this meta-analysis. Each point represents a separate study for the indicated association by T over C allele.
(DOCX)

Figure S2 **Funnel plot of publication bias in rs2910164 studies.** Each point represents a separate study for the indicated association by C over G allele.
(DOCX)

Table S1 **ORs (95% CI) of sensitivity analysis for rs11614913 and rs2910164.**
(DOCX)

## References

[1] Bartel DP (2004). MicroRNAs: genomics, biogenesis, mechanism, and function.. Cell.

[2] He L, Hannon GJ (2004). MicroRNAs: small RNAs with a big role in gene regulation.. Nat Rev Genet.

[3] Calin GA, Croce CM (2006). MicroRNA signatures in human cancers.. Nat Rev Cancer.

[4] Paranjape T, Slack FJ, Weidhaas JB (2009). MicroRNAs: tools for cancer diagnostics.. Gut.

[5] Saunders MA, Liang H, Li WH (2007). Human polymorphism at microRNAs and microRNA target sites.. Proc Natl Acad Sci U S A.

[6] Dou T, Wu Q, Chen X, Ribas J, Ni X (2010). A polymorphism of microRNA196a genome region was associated with decreased risk of glioma in Chinese population.. J Cancer Res Clin Oncol.

[7] Liu Z, Li G, Wei S, Niu J, El-Naggar AK (2010). Genetic variants in selected pre-microRNA genes and the risk of squamous cell carcinoma of the head and neck.. Cancer.

[8] Tian T, Shu Y, Chen J, Hu Z, Xu L (2009). A functional genetic variant in microRNA-196a2 is associated with increased susceptibility of lung cancer in Chinese.. Cancer Epidemiol Biomarkers Prev.

[9] Kim MJ, Yoo SS, Choi YY, Park JY (2010). A functional polymorphism in the pre-microRNA-196a2 and the risk of lung cancer in a Korean population.. Lung Cancer.

[10] Guo H, Wang K, Xiong G, Hu H, Wang D (2010). A functional varient in microRNA-146a is associated with risk of esophageal squamous cell carcinoma in Chinese Han.. Fam Cancer.

[11] Peng S, Kuang Z, Sheng C, Zhang Y, Xu H (2010). Association of microRNA-196a-2 gene polymorphism with gastric cancer risk in a Chinese population.. Dig Dis Sci.

[12] Srivastava K, Srivastava A, Mittal B (2010). Common genetic variants in pre-microRNAs and risk of gallbladder cancer in North Indian population.. J Hum Genet.

[13] Li XD, Li ZG, Song XX, Liu CF (2010). A variant in microRNA-196a2 is associated with susceptibility to hepatocellular carcinoma in Chinese patients with cirrhosis.. Pathology.

[14] Qi P, Dou TH, Geng L, Zhou FG, Gu X (2010). Association of a variant in MIR 196A2 with susceptibility to hepatocellular carcinoma in male Chinese patients with chronic hepatitis B virus infection.. Hum Immunol.

[15] Xu T, Zhu Y, Wei QK, Yuan Y, Zhou F (2008). A functional polymorphism in the miR-146a gene is associated with the risk for hepatocellular carcinoma.. Carcinogenesis.

[16] Hu Z, Liang J, Wang Z, Tian T, Zhou X (2009). Common genetic variants in pre-microRNAs were associated with increased risk of breast cancer in Chinese women.. Hum Mutat.

[17] Hoffman AE, Zheng T, Yi C, Leaderer D, Weidhaas J (2009). microRNA miR-196a-2 and breast cancer: a genetic and epigenetic association study and functional analysis.. Cancer Res.

[18] Catucci I, Yang R, Verderio P, Pizzamiglio S, Heesen L (2010). Evaluation of SNPs in miR-146a,miR196a2 and miR-499 as low-penetrance alleles in German and Italian familial breast cancer cases.. Hum Mutat.

[19] Pastrello C, Polesel J, Della Puppa L, Viel A, Maestro R (2010). Association between hsa-mir-146a genotype and tumor age-of-onset in BRCA1/BRCA2-negative familial breast and ovarian cancer patients.. Carcinogenesis.

[20] Xu B, Feng NH, Li PC, Tao J, Wu D (2010). A functional polymorphism in Pre-miR-146a gene is associated with prostate cancer risk and mature miR-146a expression in vivo.. Prostate.

[21] Jazdzewski K, Murray EL, Franssila K, Jarzab B, Schoenberg DR (2008). Common SNP in pre-miR-146a decreases mature miR expression and predisposes to papillary thyroid carcinoma.. Proc Natl Acad Sci U S A.

[22] Cochran WG (1954). The combination of estimates from different experiments.. Biometrics.

[23] Mantel N, Haenszel W (1959). Statistical aspects of the analysis of data from retrospective studies of disease.. J Natl Cancer Inst.

[24] DerSimonian R, Laird N (1986). Meta-analysis in clinical trials.. Control Clin Trials.

[25] Egger M, Davey Smith G, Schneider M, Minder C (1997). Bias in meta-analysis detected by a simple, graphical test.. BMJ.

[26] Christensen BC, Avissar-Whiting M, Ouellet LG, Butler RA, Nelson HH (2010). Mature microRNA sequence polymorphism in MIR196A2 is associated with risk and prognosis of head and neck cancer.. Clin Cancer Res.

[27] Hu Z, Chen J, Tian T, Zhou X, Gu H (2008). Genetic variants of miRNA sequences and non-small cell lung cancer survival.. J Clin Invest.

[28] Ye Y, Wang KK, Gu J, Yang H, Lin J (2008). Genetic variations in microRNA-related genes are novel susceptibility loci for esophageal cancer risk.. Cancer Prev Res (Phila).

[29] Clague J, Lippman SM, Yang H, Hildebrandt MA, Ye Y (2010). Genetic variation in MicroRNA genes and risk of oral premalignant lesions.. Mol Carcinog.

[30] Wu M, Jolicoeur N, Li Z, Zhang L, Fortin Y (2008). Genetic variations of microRNAs in human cancer and their effects on the expression of miRNAs.. Carcinogenesis.

[31] Lee HC, Kim JG, Chae YS, Sohn SK, Kang BW (2010). Prognostic impact of microRNA-related gene polymorphisms on survival of patients with colorectal cancer.. J Cancer Res Clin Oncol.

[32] Kontorovich T, Levy A, Korostishevsky M, Nir U, Friedman E (2010). Single nucleotide polymorphisms in miRNA binding sites and miRNA genes as breast/ovarian cancer risk modifiers in Jewish high-risk women.. Int J Cancer.

[33] Shen J, Ambrosone CB, DiCioccio RA, Odunsi K, Lele SB (2008). A functional polymorphism in the miR-146a gene and age of familial breast/ovarian cancer diagnosis.. Carcinogenesis.

[34] Yekta S, Shih IH, Bartel DP (2004). MicroRNA-directed cleavage of HOXB8 mRNA.. Science.

[35] Ma L, Teruya-Feldstein J, Weinberg RA (2007). Tumour invasion and metastasis initiated by microRNA-10b in breast cancer.. Nature.

