# Supplementary material for: Effects of Common Polymorphisms rs11614913 in miR-196a2 and rs2910164 in miR-146a on Cancer Susceptibility: A Meta-Analysis
Source: PLoS One. 2011 May 26;6(5):e20471. doi: 10.1371/journal.pone.0020471 (PMC3102728; doi:10.1371/journal.pone.0020471)
Supplement: Figure S2 — Funnel plot of publication bias in rs2910164 studies. Each point represents a separate study for the indicated association by C over G allele. (DOCX) [file pone.0020471.s002.docx]

Funnel plot of publication bias in rs2910164 studies. Log OR is plotted versus standard error for each of studies in this meta-analysis. Each point represents a separate study for the indicated association by C over G allele.
