# Supplementary material for: Effects of Common Polymorphisms rs11614913 in miR-196a2 and rs2910164 in miR-146a on Cancer Susceptibility: A Meta-Analysis
Source: PLoS One. 2011 May 26;6(5):e20471. doi: 10.1371/journal.pone.0020471 (PMC3102728; doi:10.1371/journal.pone.0020471)
Supplement: Table S1 — ORs (95% CI) of sensitivity analysis for rs11614913 and rs2910164. (DOCX) [file pone.0020471.s003.docx]

| **Table S1**: ORs (95% CI) of sensitivity analysis for rs11614913 and rs2910164. | | | | | |
| --- | --- | --- | --- | --- | --- |
| Excluding literature | TT *vs* CC | CT *vs* CC | T *vs* C | Dominant model | Recessive model |
| one by one | OR (95% CI) P*h* | OR (95% CI) P*h* | OR (95% CI) P*h* | OR (95% CI) P*h* | OR (95% CI) P*h* |
| All for rs11614913 | 0.92(0.85-0.99)0.45 | 0.98(0.93-1.03)0.87 | 0.96(0.92-0.99)0.61 | 0.97(0.93-1.02)0.93 | 0.90(0.84-0.97)0.50 |
| Hu 2008 | 0.93(0.85-1.00)0.38 | 0.98(0.93-1.04)0.82 | 0.96(0.92-1.00)0.55 | 0.97(0.93-1.03)0.89 | 0.91(0.84-0.99)0.43 |
| Tian 2009 | 0.92(0.85-0.99)0.36 | 0.98(0.93-1.05)0.84 | 0.96(0.92-0.99)0.52 | 0.97(0.93-1.03)0.89 | 0.90(0.83-0.97)0.42 |
| Hoffman 2009 | 0.94(0.86-1.01)0.86 | 0.98(0.93-1.04)0.83 | 0.96(0.92-1.00)0.97 | 0.98(0.93-1.03)0.93 | 0.92(0.85-0.99)0.92 |
| Srivastava 2010 | 0.92(0.85-0.99)0.37 | 0.98(0.93-1.04)0.91 | 0.96(0.92-0.99)0.54 | 0.96(0.92-1.00)0.54 | 0.90(0.84-0.97)0.46 |
| Min 2010 | 0.93(0.85-1.01)0.39 | 0.98(0.93-1.04)0.83 | 0.96(0.92-0.99)0.55 | 0.97(0.93-1.02)0.89 | 0.91(0.84-0.98)0.43 |
| Li 2010 | 0.92(0.85-1.00)0.41 | 0.98(0.93-1.04)0.82 | 0.96(0.92-0.99)0.59 | 0.97(0.93-1.02)0.89 | 0.91(0.85-0.98)0.51 |
| Dou 2010 | 0.90(0.83-0.98)0.49 | 0.97(0.91-1.03)0.93 | 0.95(0.91-0.99)0.63 | 0.96(0.92-1.01)0.96 | 0.90(0.83-0.97)0.41 |
| Qi 2010 | 0.91(0.84-0.99)0.41 | 0.98(0.92-1.04)0.82 | 0.95(0.91-0.99)0.58 | 0.97(0.92-1.02)0.89 | 0.90(0.83-0.97)0.51 |
| Catucci 2010 | 0.91(0.83-0.99)0.38 | 0.97(0.91-1.04)0.82 | 0.95(0.91-0.99)0.53 | 0.97(0.92-1.02)0.88 | 0.90(0.83-0.97)0.43 |
| Liu 2010 | 0.90(0.83-0.98)0.46 | 0.97(0.91-1.03)0.89 | 0.95(0.91-0.98)0.70 | 0.96(0.91-1.01)0.95 | 0.90(0.83-0.97)0.46 |
| Peng 2010 | 0.92(0.85-1.00)0.41 | 0.98(0.93-1.04)0.87 | 0.96(0.92-0.99)0.57 | 0.97(0.93-1.02)0.92 | 0.91(0.84-0.98)0.41 |
| Excluding literature | CC *vs* GG | CG *vs* GG | C *vs* G | Dominant model | Recessive model |
| one by one | OR (95% CI) P*h* | OR (95% CI) P*h* | OR (95% CI) P*h* | OR (95% CI) P*h* | OR (95% CI) P*h* |
| All for rs2910164 | 0.89(0.75-1.05)0.03 | 1.01(0.95-1.07)0.41 | 0.94(0.88-1.01)0.04 | 0.96(0.89-1.04)0.06 | 0.86(0.73-1.01)0.03 |
| Hu 2008 | 0.92(0.82-1.04)0.02 | 1.00(0.93-1.08)0.32 | 0.93(0.86-1.01)0.03 | 0.95(0.87-1.04)0.05 | 0.82(0.67-1.00)0.02 |
| Xu 2008 | 0.88(0.72-1.07)0.02 | 1.01(0.95-1.08)0.36 | 0.95(0.88-1.02)0.02 | 0.96(0.91-1.01)0.04 | 0.85(0.70-1.03)0.02 |
| Krystian 2008 | 0.93(0.79-1.09)0.07 | 0.98(0.92-1.04)0.96 | 0.93(0.87-1.00)0.07 | 0.94(0.88-1.01)0.27 | 0.91(0.80-1.04)0.05 |
| Tian 2009 | 0.85(0.69-1.03)0.03 | 1.00(0.94-1.07)0.32 | 0.93(0.86-1.01)0.05 | 0.96(0.89-1.04)0.06 | 0.82(0.68-1.01)0.04 |
| Srivastava 2010 | 0.90(0.76-1.07)0.03 | 1.01(0.95-1.07)0.35 | 0.95(0.88-1.02)0.03 | 0.96(0.90-1.01)0.05 | 0.87(0.74-1.02)0.03 |
| Catucci 2010 | 0.85(0.70-1.01)0.15 | 1.01(0.95-1.09)0.33 | 0.97(0.91-1.03)0.17 | 0.99(0.94-1.06)0.44 | 0.83(0.68-1.01)0.12 |
| Liu 2010 | 0.86(0.71-1.05)0.02 | 1.00(0.94-1.07)0.33 | 0.93(0.86-1.01)0.04 | 0.95(0.87-1.04)0.06 | 0.83(0.69-1.00)0.02 |
| Xu 2010 | 0.91(0.76-1.09)0.03 | 1.01(0.95-1.08)0.36 | 0.95(0.88-1.02)0.03 | 0.97(0.89-1.05)0.04 | 0.87(0.74-1.04)0.03 |
| Guo 2010 | 0.95(0.82-1.09)0.03 | 1.02(0.95-1.08)0.46 | 0.96(0.90-1.03)0.07 | 0.97(0.90-1.05)0.06 | 0.90(0.77-1.04)0.08 |
| Pastrello 2010 | 0.88(0.74-1.05)0.02 | 1.01(0.95-1.07)0.32 | 0.94(0.88-1.01)0.02 | 0.96(0.89-1.04)0.04 | 0.85(0.72-1.00)0.02 |
| P*h*: P-value of Q-test for heterogeneity test. | | |  |  |  |
